# Signals of gastroesophageal reflux disease caused by incretin-based drugs: a disproportionality analysis using the Japanese adverse drug event report database

**DOI:** 10.1186/s40780-018-0109-z

**Published:** 2018-06-18

**Authors:** Yoshihiro Noguchi, Hayato Katsuno, Anri Ueno, Manami Otsubo, Aki Yoshida, Yuta Kanematsu, Ikuto Sugita, Hiroki Esaki, Tomoya Tachi, Teruo Tsuchiya, Hitomi Teramachi

**Affiliations:** 1 Laboratory of Clinical Pharmacy, Gifu Pharmaceutical University, 1-25-4, Daigakunishi, Gifu, 501-1196 Japan; 2 Department of Pharmacy, Ichinomiya Municipal Hospita, 2-22-2 Bunkyou, Aichi, 491-8558 Japan; 3 Community Health Support and Research Center, 5-6-1 Kikuchichou, Gifu, 501-6242 Japan; 4 Laboratory of Community Healthcare Pharmacy, Gifu Pharmaceutical University, 1-25-4, Daigakunishi, Gifu, 501-1196 Japan

**Keywords:** Disproportionality analysis, Incretin-based drugs, Glucagon-like peptide-1 receptor agonists, DPP-4 inhibitors, Gastroesophageal reflux disease, Japanese adverse drug event report database

## Abstract

**Background:**

Incretin-based drugs are important in the treatment of type 2 diabetes. However, among the incretin-based drugs, glucagon-like peptide-1 receptor agonists (GLP-1-RAs) have been reported to cause gastroesophageal reflux disease (GERD)-like symptoms making it difficult to continue treatment. Therefore, with the aim of clarifying the relationship between incretin-based drugs and GERD-like symptoms, we conducted a pharmacoepidemiological study using the Japanese adverse drug event report database (JADER).

**Methods:**

Dipeptidyl peptidase-4 inhibitors (DPP-4-Is) and GLP-1-RAs were set as the incretin-based target drugs. The reporting odds ratio (ROR) and the information component (IC) was used for the detection of quantitative signals. Furthermore, we also compared the time to onset of GERD-like symptoms by log-rank test.

**Results:**

GERD-like symptoms were reported in 36 GLP-1-RAs cases (ROR: 5.61, 95% confidence interval (95% CI): 3.95–7.96 and IC: 2.17, 95% CI: 1.66–2.67) and GLP-1-RAs were detected in the signal. In contrast, DPP-4-Is were not detected in the signal.

There was no sex difference with regard to the expression time of GERD-like symptoms by GLP-1-RAs (log-rank test, *p* = 0.5381). However, the expression time of GERD-like symptoms from GLP-1-RAs was shorter in patients older than 70 years of age than that in those younger than 70 years of age (log-rank test, *p* < 0.0001).

**Conclusions:**

The administration of GLP-1-RA had a higher incidence of GERD-like symptoms earlier than the administration of DPP-4-Is. In this study, although we think that further investigation is necessary, and suggest that patients older than 70 years of age who have been administered GLP-1-RAs need earlier attention to address GERD-like symptoms than younger patients.

## Background

Incretin is a generic term for peptide hormones that have hypoglycemic action and are secreted from gastrointestinal epithelial cells with the intake of meals. Glucose-dependent insulinotropic polypeptide (GIP), which is mainly secreted by K cells distributed in the duodenum, and glucagon-like peptide-1 (GLP-1), secreted by L cells distributed in the ileum and colon, are known as incretin. These incretins promote insulin secretion from pancreatic β cells in a blood sugar-dependent manner.

At present, incretin-based drugs that can be used in a clinical setting include DPP-4 inhibitors (DPP-4-Is) that inhibit dipeptidyl peptidase-4 (DPP-4, an incretin degrader) and GLP-1 receptor agonists (GLP-1-RAs) which are not susceptible to degradation by DPP-4.

GLP-1 has an inhibitory action on the glucagon secretion of pancreatic α cells and an appetite-suppressing action through the central nervous system. Therefore, GLP-1-RAs are more effective than conventional sulfonylurea drugs and glinide drugs (conventional insulin secretagogues) for treating type 2 diabetes, in which hypoglycemia and increased body weight are less likely to occur and in which insulin secretion is reduced.

Unfortunately, it has been reported that gastroesophageal reflux disease (GERD)-like symptoms such as vomiting, nausea, impaired gastric emptying, and oesophageal ulcer can appear during GLP-1-RA treatment, making treatment continuity difficult [1]. For this reason, risk assessments on the GERD-like symptoms of GLP-1-RA and DPP-4-I would be beneficial in drug selection and to provide information when treating diabetes. However, there are few reports assessing the risk of GERD-like symptoms from incretin-based drugs in Japan [2].

Safety signals based on the principle of disproportionality and focusing on the differences in the number of reports of adverse events are used for the safety assessment of drugs after marketing [3–7]. Data mining algorithms for the quantitative detection of signals from large databases include the proportional reporting ratio (PRR) [8], the reporting odds ratio (ROR) [9], the information component (IC) [10], and the empirical Bayes geometric mean (EBGM) [11]. In addition to these detection methods, there is also a method using association rule mining and signal value [12–14].

PRR and ROR are easy to calculate, but if the number of reports is small, the signal is unstable. IC and EBGM involve calculating the parameters of the prior distribution as well, lengthening the calculation of the signal, but once calculated, the signal is thought to be relatively stable even if the number of reports is small [15]. Using association rule mining, a signal considering various factors can be easily detected, but since it is a frequency-theoretic statistical method, if the number of reports is small, the signal may not be stabilized.

There are reports that the signal value [12] proposed by Takagi et al. is used as a method of comparing signals between two groups [16].

In addition to these signals, Takada et al. proposed a method to detect inverse association between drugs and adverse events using disproportionality analysis and sequence symmetry analysis. For detect inverse association between drug and ADR, ROR and IC are used in disproportionality analysis. Detection criteria were upper limits of the 95% confidence interval (CI) of < 1 and < 0 for the ROR and IC. It is combined with the result of sequence symmetry analysis using the claim database as a guarantee of the reliability of the inverse association signal [17].

Therefore, in this study using the Japanese adverse drug event report (JADER) database published by the Pharmaceuticals and Medical Devices Agency, as the Japanese regulatory authority, we calculated the ROR and IC of GERD-like symptoms induced by incretin-based drugs and also reported on sex differences and the influence of age on the occurrence of these symptoms.

## Methods

### Data sources

The JADER database consists of four data sets: patient demographic information, drug information, adverse event information, and primary disease information.

In this study, data from diabetic patients registered in JADER from the first quarter of 2004 to the fourth quarter of 2015 were used. However, reports with missing information on sex, age, or primary disease, and where subjective terms such as “youth” and “elderly” were used, were excluded from the analysis data. Therefore, the total number of cases that could be used for analysis was 38,887 cases. The details of the cases used for analysis are shown in Table 1.

**Table 1:** The details of the cases used for analysis

	GERD-like symptoms	Total
Total	654	38,887
Sex		
Male	363	23,669
Female	291	15,218
Age		
–9	0	40
10–19	1	86
20–29	3	215
30–39	11	748
40–49	39	1990
50–59	85	5115
60–69	204	11,399
70–79	213	13,178
80–89	87	5568
90–	11	548

*GERD* gastroesophageal reflux disease

### Definitions of suspect drugs and adverse events

The target drugs were GLP-1-RAs and DPP-4-Is. (1) The GLP-1-RAs included dulaglutide, exenatide, liraglutide and lixisenatide. (2) The DPP-4-Is included alogliptin, anagliptin, linagliptin, omarigliptin, saxagliptin, sitagliptin, teneligliptin, trelagliptin, teneligliptin, trelagliptin and vildagliptin.

Because the standardized Medical Dictionary for Regulatory Activities does not have a set definition for GERD-like symptoms, they were defined as 36 preferred terms (PTs) as shown in Table 1.

### Signal detection

We performed a disproportionality analysis, which can be generally viewed as a case/non-case analysis. The cases were defined as GERD-like symptoms in target adverse events; the non-cases were defined as all adverse events without GERD-like symptoms.

The number of reports of adverse events due to the use of the target drugs was counted based on the number of cases.

Safety signal as a measure of disproportionality, the ROR, the IC, and their 95% confidence interval (95% CI) were calculated [10].

In addition, the relevance of differences in sex and age was investigated in this context.

Female are many items to evaluate as before and after menopause, pregnant and lactating, but JADER does not include any data. Moreover, it is difficult to decide and evaluate them only by age. Therefore, in this study, we made a simple evaluation with male vs. female.

The age reported in JADER is provided as data separated every 10 years for the sake of privacy consideration. Generally, it is generally that elderly people are defined as being over 65 years old. However, since there is an opinion that the definition of elderly should be raised to a higher age reflecting Japanese longevity and aging, the age categories used were patients older than 70 years and patients younger than 70 years.

The signal values were used as a method to compare signals between the two groups (differences in sex or age). Signal values to be compared were calculated from the PRR and chi-squared test (χ2) values of 2 groups, respectively, using formula (1) proposed by Takagi et al. [12]:

1 $$ \mathrm{Signal}\ \mathrm{value}=\ln \left(\mathrm{PRR}\right)+\ln \left({\upchi}^2\right) $$

The detection criteria of the signal value is shown in the following formula (2) using 2 groups of patients older than 70 years and patients younger than 70 years as an example [16]:

(signal value of patients older than 70 years) > 2(signal value of patients younger than 70 years) ^…^(2).

### Comparison of the onset time profile

Log-rank tests were conducted to assess the relationship between DPP-4-I and GLP-1-RA, and significant differences in sex and age, for GERD-like symptoms expressed within 1 year of incretin-based drug administration.

In this study, the number of days from the administration start date of the drug to the occurrence date of the adverse event was used as the time to onset. For non-cases this was the number of days until the end of drug use. A maximum of 365 days was used as the discontinuation date for those with a period of 1 year or more before onset.

### Statistical analysis software

We used visual mining studio (version 8.2, NTT Mathematical System, Tokyo, Japan) for Signal detection, JMP (version 11, SAS, NY, USA) for drawing Kaplan-Meier curves (GERD-like symptoms expression time curves) and conducting log-rank tests.

## Results

### Signal detection

There were 36 reported cases of GLP-1-RA-related GERD-like symptoms (ROR: 5.61, 95% CI: 3.95–7.96 and IC: 2.17, 95% CI: 1.66–2.67) and GLP-1-RAs were detected in the signal.

The individual signal values of drugs classified as GLP-1-RAs were as follows: dulaglutide (ROR: 3.08, 95% CI: 0.41–23.04 and IC: 0.56, 95% CI: -1.57 – 2.70), exenatide (ROR: 11.08, 95% CI: 6.75–18.18 and IC: 2.70, 95% CI: 2.00–3.41), liraglutide (ROR: 3.39, 95% CI: 2.00–5.74 and IC: 1.49, 95% CI: 0.74–2.24), lixisenatide (ROR: 5.32, 95% CI: 0.69–41.28 and IC: 0.71, 95% CI: -1.48 – 2.91), and both signals were detected in exenatide and liraglutide (Table 2).

**Table 2:** The preferred term identifier and name included in GERD-like symptoms

PT ID	PT name	PT ID	PT name
10000059	Abdominal discomfort	10053634	Oesophageal discomfort
10000060	Abdominal distension	10065835	Oesophageal fistula
10075494	Acid peptic disease	10030172	Oesophageal haemorrhage
10007645	Cardiospasm	10070818	Oesophageal irritation
10013924	Dyskinesia oesophageal	10072280	Oesophageal mucosa erythema
10013946	Dyspepsia	10030178	Oesophageal obstruction
10013950	Dysphagia	10030180	Oesophageal pain
10063655	Erosive oesophagitis	10030181	Oesophageal perforation
10052405	Gastric hypomotility	10052211	Oesophageal rupture
10017885	Gastrooesophageal reflux disease	10030194	Oesophageal stenosis
10062879	Gastrooesophageal sphincter insufficiency	10030201	Oesophageal ulcer
10021518	Impaired gastric emptying	10030202	Oesophageal ulcer haemorrhage
10028813	Nausea	10052488	Oesophageal ulcer perforation
10055668	Necrotising oesophagitis	10030216	Oesophagitis
10062501	Non-cardiac chest pain	10030219	Oesophagitis haemorrhagic
10030136	Oesophageal achalasia	10056992	Oesophagobronchial fistula
10000059	Abdominal discomfort	10072163	Oesophagogastroduodenoscopy abnormal
10000060	Abdominal distension	10072166	Oesophagogastroscopy abnormal
10075494	Acid peptic disease	10030223	Oesophagoscopy abnormal
10007645	Cardiospasm	10047700	Vomiting

Among the adverse events (PTs) investigated as GERD-like symptoms, the PTs reported for GLP-1-RA were vomiting, nausea, impaired gastric emptying, oesophageal ulcer, gastrooesophageal reflux disease, dysphagia, and abdominal distension. Significant 2 signals (both ROR and IC) were detected for vomiting (ROR: 6.73, 95% CI: 4.36–10.39 and IC: 2.32, 95% CI: 1.69–2.94), nausea (ROR: 5.37, 95% CI: 3.26–8.85 and IC: 2.02, 95% CI: 1.31–2.73), significant 1 signal (ROR only) were detected for impaired gastric emptying (ROR: 44.93, 95% CI: 4.07–496.40 and IC: 0.94, 95% CI: -1.57 – 3.44) (Table 3).

**Table 3:** The signal values of GERD-like symptoms of GLP-1-RAs and DPP-4-Is

Class	Drug name	n_11_	n_1+_	ROR (95%CI)	IC (95%CI)
GLP-1-RAs	(ALL)	36	429	5.61* (3.95–7.96)	2.17* (1.66–2.67)
	dulaglutide	1	20	3.08 (0.41–23.04)	0.56 (−1.57–2.70)
	exenatide	19	122	11.08* (6.75–18.18)	2.70* (2.00–3.41)
	liraglutide	15	278	3.39* (2.00–5.74)	1.49* (0.74–2.24)
	lixisenatide	1	12	5.32 (0.69–41.28)	0.71 (− 1.48–2.91)
DPP-4-Is	(ALL)	31	3276	0.54 (0.37–0.77)	-0.81 (− 1.34 – −0.29)
	alogliptin	1	345	0.17 (0.02–1.20)	− 1.77 (−3.82–0.28)
	anagliptin	0	72	–	–
	linagliptin	4	332	0.71 (0.26–1.91)	−0.40 (−1.71–0.90)
	omarigliptin	0	2	–	–
	saxagliptin	0	70	–	–
	sitagliptin	16	1181	0.80 (0.48–1.31)	−0.30 (−1.01–0.41)
	teneligliptin	0	98	–	–
	trelagliptin	1	19	3.25 (0.43–24.39)	0.58 (−1.56–2.72)
	vildagliptin	9	1189	0.44 (0.23–0.85)	−1.07 (−2.00 – −0.15)

*GERD* gastroesophageal reflux disease, *GLP-1-RA*s glucagon-like peptide-1 receptor agonists, *DDP*-4-*Is* dipeptidyl peptidase-4 inhibitors, *n*_*11*_ the number of target drug induced GERD-like symptoms, *n*_*1+*_ the number of target drug induced all adverse events, *ROR* reporting odds ratio, *IC* information component

* signal detection

In contrast, although there were 31 reports of GERD-like symptoms, DPP-4-Is were not detected in the signal (ROR: 0.54, 95% CI: 0.37–0.77 and IC: -0.81, 95% CI: -1.34 – -0.29) (Table 2).

Among the adverse events (PTs) investigated as GERD-like symptoms, the PTs reported for DPP-4-Is were vomiting, nausea, impaired gastric emptying, oesophageal ulcer, gastrooesophageal reflux disease, dysphagia, abdominal distension, erosive oesophagitis, dyspepsia. However, no significant 2 signals (both ROR and IC) were detected (Tables 3 and 4).

**Table 4:** The signal value per preferred term of GERD-like symptoms of GLP-1-RAs and DPP-4-Is

AE	GLP-1-RAs (n_1+_ = 429)				DPP-4-Is (n_1+_ = 3276)			
	n_11_	n _+ 1_	ROR (95%CI)	IC (95%CI)	n_11_	n _+ 1_	ROR (95%CI)	IC (95%CI)
Vomiting	23	344	6.73* (4.36–10.39)	2.32* (1.69–2.94)	12	344	0.39 (0.22–0.70)	−1.21 (−2.03 – −0.39)
Nausea	17	310	5.37* (3.26–8.85)	2.02* (1.31–2.73)	9	310	0.32 (0.17–0.63)	−1.44 (− 2.37 – − 0.52)
Impaired gastric emptying	1	3	44.93* (4.07–496.40)	0.94 (− 1.57–3.44)	0	3	–	–
Oesophageal ulcer	1	18	5.28(0.70–39.79)	0.72 (− 1.42–2.87)	0	18	–	–
Gastrooesophageal reflux disease	1	24	3.90 (0.53–28.98)	0.65 (− 1.48–2.77)	6	18	3.63* (1.44–9.15)	1.17 (− 0.06–2.41)
Dysphagia	1	28	3.33 (0.45–24.53)	0.60 (−1.52–2.71)	5	28	2.37 (0.90–6.23)	0.80 (−0.49–2.10)
Abdominal distension	1	33	2.81 (0.38–20.58)	0.54 (− 1.56–2.64)	1	33	0.34 (0.05–2.49)	−0.95 (−3.05–1.15)
Erosive oesophagitis	0	25	–	–	1	25	0.45 (0.06–3.35)	− 0.67 (− 2.79–1.44)
Dyspepsia	0	45	–	–	1	45	0.25 (0.03–1.79)	−1.29(− 3.37–0.80)

*AE* adverse event, *GERD* gastroesophageal reflux disease, *GLP-1-RAs* glucagon-like peptide-1 receptor agonists, *DDP-4-Is* dipeptidyl peptidase-4 inhibitors, *n11* the number of target drug induced GERD-like symptoms, *n1+* the number of target drug induced all adverse events, *n + 1* the number of all drug induced target adverse events, *ROR* reporting odds ratio, *IC* information component

* signal detection

The signal of males taking GLP-1-RAs was ROR: 6.86, 95% CI: 4.37–10.78 and IC: 2.29, 95% CI: 1.65–2.94, females’ signal was ROR: 4.24, 95% CI: 2.43–7.39 and IC: 1.69, 95% CI: 0.89–2.48, and signals were detected in both sexes. The signal values were 6.33 for males and 4.71 for females. From this result, sex difference was not observed.

The signal of patients older than 70 years taking GLP-1-RAs was ROR: 10.43, 95% CI: 6.68–16.29 and IC: 2.70, 95% CI: 2.06–3.34, the signal of patients younger than 70 years was ROR: 2.85, 95% CI: 1.58–5.14 and IC: 1.25, 95% CI: 0.41–2.08, and signals were detected in both age groups. The signal values were 7.26 for patients older than 70 years and 3.46 for patients younger than 70 years. From this result, compared with patients younger than 70 years, strong association was shown to patients older than 70 years.

### Comparison of the time to onset profile

**Fig. 1:**
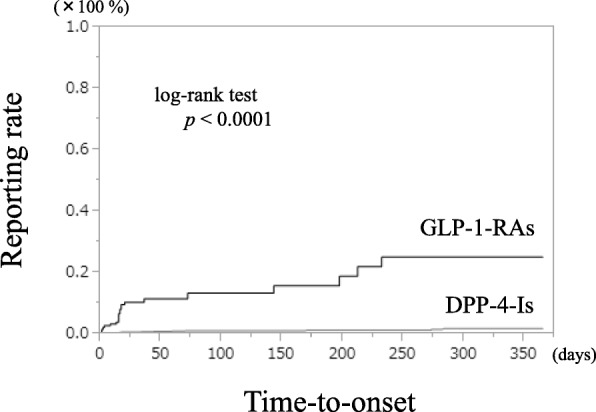
Kaplan-Meier curve of GERD-like symptom manifestation from GLP-1-RAs and DPP-4-Is. ※GERD: gastroesophageal reflux disease, GLP-1-RAs: glucagon-like peptide-1 receptor agonists, DDP-4-Is: dipeptidyl peptidase-4 inhibitors

Figure 1 shows the GERD-like symptom expression time curves of GLP-1-RAs and DPP-4-Is. After excluding reports with a lack of time series data, 26 out of 36 (72.2%) reports of GLP-1-RAs-related GERD-like symptoms reported symptoms that developed within 1 year. In contrast, after similar exclusions, 19 out of 31 (61.3%) reports of DPP-4-Is-related GERD-like symptoms reported symptoms that developed within 1 year.

In 307 GLP-1-RAs cases (71.6%) and 1873 DPP-4-Is cases (57.2%) with no missing time series data, administration ceased due to the onset of adverse events other than GERD-like symptoms. The onset of GERD-like symptoms in the GLP-1-RAs in which signal was detected tended to be significantly faster than that in the DPP-4-Is in which no signal was detected (log-rank test *p* < 0.0001).

**Fig. 2:**
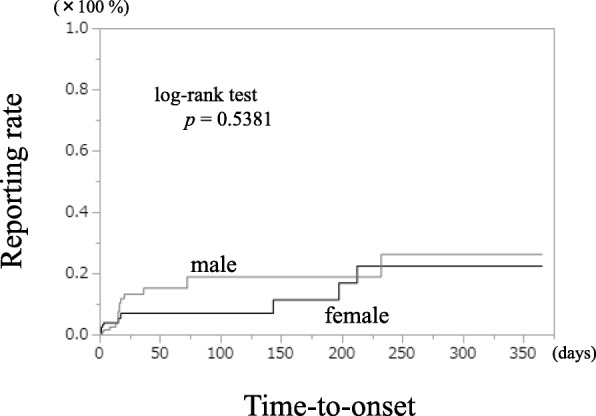
Kaplan Meier curve of GERD-like symptom manifestation from GLP-1-RAs (male vs. female). ※GERD: gastroesophageal reflux disease, GLP-1-RAs:glucagon-like peptide-1 receptor agonists

Figure 2 shows the male and female expression time curves of GERD-like symptoms from GLP-1-RAs. There was no sex-related difference in symptom expression time (log-rank test *p* = 0.5381).

**Fig. 3:**
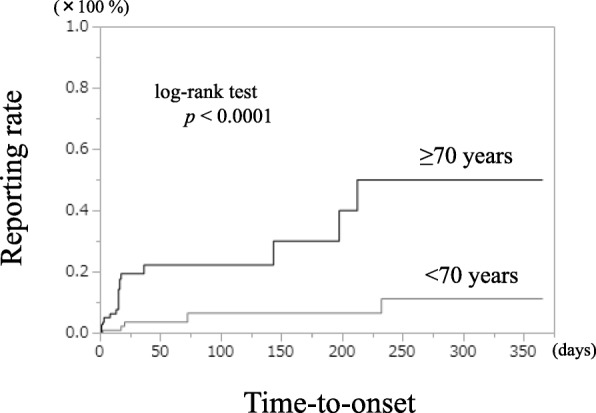
Kaplan Meier curve of GERD-like symptom manifestation from GLP-1-RAs (patients older than 70 years vs. patients younger than 70 years). ※GERD: gastroesophageal reflux disease, GLP-1-RAs:glucagon-like peptide-1 receptor agonists

Figure 3 shows the expression time curves for patients older than 70 years and patients younger than 70 years old of GERD-like symptoms from GLP-1-RAs. The expression time was earlier in patients older than 70 years of age (log-rank test *p* < 0.0001).

## Discussion

In this study, we focused on the GERD-like symptoms of incretin drug usage and evaluated the risk of incretin-based GLP-1-RAs and DPP-4-Is using JADER. Furthermore, we investigated the effects of sex and age on the onset of symptoms from GLP-1-RAs.

Among the incretin-based drugs, there were 2 GLP-1-RAs (exenatide, liraglutide) for which signals of GERD-like symptoms were detected. In comparison, all DPP-4-Is were not detected.

GERD was defined as “a condition that develops when the reflux of stomach contents causes troublesome symptoms and/or complications” [18].

In this study, as the term “GERD” was not listed in the Standard MedDRA Queries, 36 PTs considered to be applicable (Table 1) were defined as GERD-like symptoms.

Among the 36 PTs constituting GERD-like symptoms, PTs reported in the use of GLP-1-RAs were vomiting, nausea, impaired gastric emptying, esophageal ulcer, gastroesophageal reflux disease, dysphagia, and abdominal distension. These results indicate the possibility of developing GERD-like symptoms when using GLP-1-RAs. The timing of the expression of symptoms was also significantly faster in GLP-1-RAs than it was in DPP-4-Is.

It could be considered that these differences may be due to differences in the strength of pharmacological action between GLP-1-RA and DPP-4-I. DPP-4-I has similar effects to those of GLP-1, but DPP-4-I physiologically increases the level of both GIP and GLP-1. The resultant levels of GLP-1 and GIP only slightly reduce gastric peristalsis [19, 20]. However, since GLP-1-RA is peripherally administered, it is considered to have a much stronger gastric peristalsis-suppressing action than that of DPP-4-I [21]. The differences in the intensity of suppression of gastric peristalsis due to the differences in the pharmacological effects of GLP-1-RA and DPP-4-I may affect the expression of GERD-like symptoms.

In “all DPP-4-Is”, not only the signal of GERD was not detected, but a signal of inverse correlation with GERD was detected. Rather, this result suggests that DPP-4-Is may prevent GERD. However, Takada et al., who proposed the signal detection of the inverse association [17], did not conclude with only the signal of the inverse correlation obtained from the spontaneous reporting system. For this result, we could have to analyze in detail using other databases such as claim database.

Among patients using GLP-1-RAs, signals were stratified based on sex, and as a result, signals were detected in both males and females. Signals were similar between males and females, and there was no effect of sex on the timing of expression.

In the stratified analysis between patients older than 70 years and patients younger than 70 years signals were detected in both cases, but the signal value was higher in patients older than 70 years. The time of onset was also significantly faster for patients older than 70 years. In older patients it is thought that gastric juices and stomach contents could easily flow back into the esophagus, due to age-related deterioration of the contraction ability and tension of the esophagus, and that this could be further enhanced by the action of GLP-1-RAs. Therefore, when using GLP-1-RAs in patients older than 70 years, it is necessary to carefully consider the possibility of GERD-like symptoms and, if necessary, to use combination therapies such as gastrointestinal peristalsis promoters and proton pump inhibitors [2].

Since the medical database used in this study is based on spontaneous reports besides clinical trial, only the portion of adverse events recognized in clinical practice has been reported on. Furthermore, in addition to this underreporting, there are several other reporting biases such as the notoriety effect (the overall rise in the reported number of topical adverse events), and the ripple effect (along with the adverse events of specific drugs, there is an increase in the reported number of allogeneic drugs) [22].

In addition to the spontaneous reporting methods, this database also contains the results of post-marketing surveillance. Sometimes the reported number of AEs tends to decrease with the passage of time after a transient rise immediately after marketing. This reporting bias is called the Weber effect [23]. Thus, spontaneous report databases such as JADER have the disadvantage of several reporting biases. Therefore, it is necessary to pay close attention to signal interpretation.

There are many reports [24–27] of disproportionality analysis in Japan using PRR or ROR which are easier to calculate, but these methods are said to be unstable when the number of reports is small [15]. In this study, it was predicted that the number of reports would be reduced by limiting the subjects to be studied to diabetic patients and by calculating the signal for each medicine. For this reason, we also evaluated the data using IC, which is said to give more stable signals even if the number of reports is small.

This approach showed that, among the incretin-based drugs, GLP-1-RAs may be particularly associated with GERD-like symptoms. However, there are numerous confounding factors that influence GERD-like symptoms, such as the action time of each drug, and there are limitations to conducting a more detailed analysis using only the number of reports currently registered in the database. Female are many items to evaluate as before and after menopause, pregnant and lactating women, but JADER does not include any data. Therefore, as for sex difference in consideration of pregnancy women, lactation women, and before and after menopause of women, it is difficult to analyze in JADER.

Since the term “GERD” is not described in the standard MedDRA query in this study, 36 PTs considered to be applicable (Table 1) were defined as GERD-like symptoms. And detected the signal in GLP-1-RAs. However, among PT of GERD-like symptoms, in the PT of “GERD” signal could not be detected with GLP-1-RAs (Table 3). Therefore, GERD-like symptoms cannot be concluded as GERD.

## Conclusion

This study is the first to use disproportionality analysis on incretin-based drugs induced GERD-like symptom using data from a spontaneous reporting system in Japan. This result might further strengthen the evidence of previous studies. The administration of GLP-1-RAs, compared to that of DPP-4-Is, showed a higher incidence of GERD-like symptoms. Although there were no sex differences in the onset time of GERD-like symptoms from GLP-1-RAs, we suggest that patients older than 70 years who have been administered GLP-1-RAs need earlier attention to address GERD-like symptoms than younger patients.

## Abbreviations

CI: Confidence interval
DPP-4: Dipeptidyl peptidase-4
DPP-4-I: Dipeptidyl peptidase-4 inhibitor
EBGM: Empirical Bayes geometric mean
GERD: Gastroesophageal reflux disease
GIP: Glucose-dependent insulinotropic polypeptide
GLP-1: glucagon-like peptide-1
GLP-1-RA: Glucagon-like peptide-1 receptor agonist
IC: Information component
JADER: Japanese adverse drug event report database
ROR: Reporting odds ratio
PRR: Proportional reporting ratio
PT: Preferred term

## References

[1] Suzuki Y (2012). The possible high risk of GERD in healthy Asian if over dose of GLP-1 receptor agonist is injected. Jpn J Med Pharm Sci.

[2] Fushimi N, Mori A, Takeishi S, Hachiya H, Yumura T, Ohashi N, Kawai H (2014). Effects of incretin-related drugs on the incidence of gastroesophageal reflux disease-like symptoms. J Japan Diab Soc.

[3] Bate A, Evans SJ (2009). Quantitative signal detection using spontaneous ADR reporting. Pharmacoepidemiol Drug Saf.

[4] Hauben M, Reich L (2004). Drug-induced pancreatitis: lessons in data mining. Br J Clin Pharmacol.

[5] Almenoff J, Tonning JM, Gould AL, Szarfman A, Hauben M, Ouellet-Hellstrom R, Ball R, Hornbuckle K, Walsh L, Yee C, Sacks ST, Yuen N, Patadia V, Blum M, Johnston M, Gerrits C, Seifert H, Lacroix K (2005). Perspectives on the use of data mining in pharmaco-vigilance. Drug Saf.

[6] Almenoff JS, Pattishall EN, Gibbs TG, DuMouchel W, Evans SJ, Yuen N (2007). Novel statistical tools for monitoring the safety of marketed drugs. Clin Pharmacol Ther.

[7] Hauben M, Bate A (2009). Decision support methods for the detection of adverse events in post-marketing data. Drug Discov Today.

[8] Evans SJ, Waller PC, Davis S (2001). Use of proportional reporting ratios (PRRs) for signal generation from spontaneous adverse drug reaction reports. Pharmacoepidemiol Drug Saf.

[9] van Puijenbroek EP, Bate A, Leufkens HG, Lindquist M, Orre R, Egberts AC (2002). A comparison of measures of disproportionality for signal detection in spontaneous reporting systems for adverse drug reactions. Pharmacoepidemiol Drug Saf.

[10] Bate A, Lindquist M, Edwards IR, Olsson S, Orre R, Lansner A, De Freitas RM (1998). A Bayesian neural network method for adverse drug reaction signal generation. Eur J Clin Pharmacol.

[11] Szarfman A, Machado SG, O'Neill RT (2002). Use of screening algorithms and computer systems to efficiently signal higher-than-expected combinations of drugs and events in the US FDA's spontaneous reports database. Drug Saf.

[12] Shirakuni Y, Okamoto K, Kawashita N, Yasunaga T, Takagi T (2009). Signal detection of drug complications applying association rule learning for Stevens-Johnson syndrome. J Com Aid Chem.

[13] Noguchi Y, Ueno A, Otsubo M, Katsuno H, Sugita I, Kanematsu Y, Yoshida A, Esaki H, Tachi T, Teramachi H (2018). A New Search Method Using Association Rule Mining for Drug-Drug Interaction Based on Spontaneous Report System. Front Pharmacol.

[14] Noguchi Y, Ueno A, Otsubo M, Katsuno H, Sugita I, Kanematsu Y, Yoshida A, Esaki H, Tachi T, Teramachi H. A simple method for exploring adverse drug events in patients with different primary diseases using spontaneous reporting system, BMC Bioinformatics, 2018;19:124. doi: org/ 10.1186/s12859-018-2137-y,2018. 10.1186/s12859-018-2137-y PMC5887208 29621976

[15] Fujita T (2009). Signal detection of adverse drug reactions. Jpn J Pharmacoepidemiol.

[16] Noguchi Y, Ueno A, Katsuno H, Otsubo M, Yoshida A, Kanematsu Y, Sugita I, Tachi T, Tsuchiya T, Teramachi H. Analyses of non-benzodiazepine-induced adverse events and prognosis in elderly patients based on the Japanese adverse drug event report Database. J. Pharm. Health Care Sci. 2018:4, 10. doi.org/10.1186/s40780-018-0106-2,2018 10.1186/s40780-018-0106-2 PMC5937044 29760940

[17] Takada M, Fujimoto M, Motomura H, Hosomi K (2016). Inverse association between sodium channel-blocking antiepileptic drug use and cancer: data mining of spontaneous reporting and claims databases. Int J Med Sci.

[18] Vakil N, van Zanten SV, Kahrilas P, Dent J, Jones R (2006). The Montreal definition and classification of gastroesophageal reflux disease: a global evidence-based consensus. Am J Gastroenterol.

[19] Salehi M, Vahl TP, D'Alessio DA (2008). Regulation of islet hormone release and gastric emptying by endogenous glucagon-like peptide 1 after glucose ingestion. J Clin Endocrinol Metab.

[20] Drucker DJ, Nauck MA (2006). The incretin system: glucagon-like peptide-1 receptor agonists and dipeptidyl peptidase-4 inhibitors in type 2 diabetes. Lancet.

[21] Imeryüz N, Yeğen BC, Bozkurt A, Coşkun T, Villanueva-Peñacarrillo ML, Ulusoy NB (1997). Glucagon-like peptide-1 inhibits gastric emptying via vagal afferent-mediated central mechanisms. Am J Phys.

[22] Pariente A, Gregoire F, Fourrier-Reglat A, Haramburu F, Moore N (2007). Impact of safety alerts on measures of disproportionality in spontaneous reporting databases: the notoriety bias. Drug Saf.

[23] Weber J (1984). Epidemiology of adverse reactions to nonsteroidal anti-inflammatory drugs. Adv Inflamm Res.

[24] Inose R, Hosomi K, Park B, Fujimoto M, Takada M (2014). Signal detection of serious infections following treatment with biologics for rheumatoid arthritis: Data Mining of the FDA Adverse Event Reporting System and Japanese Adverse Drug Event Report Databases. Jpn J Pharm Health Care Sci.

[25] Noguchi Y, Saito K, Esaki H, Usui K, Kato M, Tachi T, Teramachi H (2016). Examination for safety of antiplatelet therapy in the elderly patients using data Mining of the Japanese Adverse Drug Event Report (JADER) database. Jpn J Drug Inform.

[26] Kose E, Uno K, Hayashi H (2017). Evaluation of the expression profile of extrapyramidal symptoms due to antipsychotics by data Mining of Japanese Adverse Drug Event Report (JADER) database. Yakugaku Zasshi.

[27] Ohyama K, Kawakami H, Inoue M (2017). Blood pressure elevation associated with topical prostaglandin F2α analogs: an analysis of the different spontaneous adverse event report databases. Biol Pharm Bull.

